# The clinical impact of CYP2C19 *2 and *3 polymorphism on Clopidogrel and cost analysis

**DOI:** 10.1186/1471-2164-15-S2-P19

**Published:** 2014-04-02

**Authors:** Al Meman A, Khalaf H, Rasool Seemab

**Affiliations:** 1 Intervention group at Prince Sultan Cardiac Center, Qassim & Genetic laboratory at Qassim University, Kingdom of Saudi Arabia

## Background

Inter individual variability to Clopidogrel response is well recognized as a strapping barrier in front of clinicians. It has been mentioned that the pharmacogenetic variations may explain 30-50 percent in this variability [1,2]. Several recent landmark studies have proven the importance of 2C19 genotyping in treatment using clopidogrel [1-3]. Therefore we sought to investigate the variability in PFT response to CYP2C19 polymorphism and compare it with the clinical presentation. Furthermore, cost analysis is considered.

## Materials and methods

One hundred and seventy Saudi patients who were stable on 75 mg clopidogrel at least for one month for various cardiac indications were enrolled in Prince Sultan Cardiac Center, Buraidah. DNA was extracted by MagNAPure [LC,Roche, Germany].CYP2C19 Genotyping for *1, *2 and *3 was conducted by real-time PCR [Roche Molecular Biochemicals, Mannheim, Germany]. PFT was carried out by (Verify Now P2Y12) for all the patients. The clinical events were documented retrospectively for all patients.

## Results

One hundred seventeen patients presented with the wild variant 1/1, 19 patients with 1/2, and 34 patients with 2/2. We couldn’t detect allele 3. There is a significant association between different genotyping and the percentage of inhibition (P = 0.0002). 1/1 patients tend to be moderate to - extensive metabolizers, whereas the majority of the rest are slow metabolizers. One hundred twenty three patients presented with no symptoms. Interestingly, no association was found between the adequacy of the PFT and the clinical symptoms (P = 0.60) and PPIs did not affect the overall response. Furthermore, Aspirin dose didn’t impact on the clinical symptoms (P = 0.080). The cost of doing the PFT is 250 SR/patient and 200 SR/patient (real-time PCR) for kits and materials excluding equipment and labors.

## Conclusions

Although the platelet function testing varied significantly with polymorphism, the clinical symptoms were not associated. The running cost is affordable if clinical association was found to be significant.
